# Intracellular Reverse Transcription of Pfizer BioNTech COVID-19 mRNA Vaccine BNT162b2 In Vitro in Human Liver Cell Line

**DOI:** 10.3390/cimb44030073

**Published:** 2022-02-25

**Authors:** Markus Aldén, Francisko Olofsson Falla, Daowei Yang, Mohammad Barghouth, Cheng Luan, Magnus Rasmussen, Yang De Marinis

**Affiliations:** 1 Department of Clinical Sciences, Lund University, 20502 Malmö, Sweden; ma7440al-s@student.lu.se (M.A.); francisko.olofsson@gmail.com (F.O.F.); daowei.yang@med.lu.se (D.Y.); mohammad.barghouth@med.lu.se (M.B.); cheng.luan@med.lu.se (C.L.); 2 Infection Medicine, Department of Clinical Sciences, Lund University, 22362 Lund, Sweden; magnus.rasmussen@med.lu.se

**Keywords:** COVID-19 mRNA vaccine, BNT162b2, liver, reverse transcription, LINE-1, Huh7

## Abstract

Preclinical studies of COVID-19 mRNA vaccine BNT162b2, developed by Pfizer and BioNTech, showed reversible hepatic effects in animals that received the BNT162b2 injection. Furthermore, a recent study showed that SARS-CoV-2 RNA can be reverse-transcribed and integrated into the genome of human cells. In this study, we investigated the effect of BNT162b2 on the human liver cell line Huh7 in vitro. Huh7 cells were exposed to BNT162b2, and quantitative PCR was performed on RNA extracted from the cells. We detected high levels of BNT162b2 in Huh7 cells and changes in gene expression of long interspersed nuclear element-1 (LINE-1), which is an endogenous reverse transcriptase. Immunohistochemistry using antibody binding to LINE-1 open reading frame-1 RNA-binding protein (ORFp1) on Huh7 cells treated with BNT162b2 indicated increased nucleus distribution of LINE-1. PCR on genomic DNA of Huh7 cells exposed to BNT162b2 amplified the DNA sequence unique to BNT162b2. Our results indicate a fast up-take of BNT162b2 into human liver cell line Huh7, leading to changes in LINE-1 expression and distribution. We also show that BNT162b2 mRNA is reverse transcribed intracellularly into DNA in as fast as 6 h upon BNT162b2 exposure.

## 1. Introduction

Coronavirus disease 2019 (COVID-19) caused by severe acute respiratory syndrome coronavirus 2 (SARS-CoV-2) was announced by the World Health Organization (WHO) as a global pandemic on 11 March 2020, and it emerged as a devasting health crisis. As of February 2022, COVID-19 has led to over 430 million reported infection cases and 5.9 million deaths worldwide [1]. Effective and safe vaccines are urgently needed to reduce the morbidity and mortality rates associated with COVID-19.

Several vaccines for COVID-19 have been developed, with particular focus on mRNA vaccines (by Pfizer-BioNTech and Moderna), replication-defective recombinant adenoviral vector vaccines (by Janssen-Johnson and Johnson, Astra-Zeneca, Sputnik-V, and CanSino), and inactivated vaccines (by Sinopharm, Bharat Biotech and Sinovac). The mRNA vaccine has the advantages of being flexible and efficient in immunogen design and manufacturing, and currently, numerous vaccine candidates are in various stages of development and application. Specifically, COVID-19 mRNA vaccine BNT162b2 developed by Pfizer and BioNTech has been evaluated in successful clinical trials [2,3,4] and administered in national COVID-19 vaccination campaigns in different regions around the world [5,6,7,8].

BNT162b2 is a lipid nanoparticle (LNP)–encapsulated, nucleoside-modified RNA vaccine (modRNA) and encodes the full-length of SARS-CoV-2 spike (S) protein, modified by two proline mutations to ensure antigenically optimal pre-fusion conformation, which mimics the intact virus to elicit virus-neutralizing antibodies [3]. Consistent with randomized clinical trials, BNT162b2 showed high efficiency in a wide range of COVID-19-related outcomes in a real-world setting [5]. Nevertheless, many challenges remain, including monitoring for long-term safety and efficacy of the vaccine. This warrants further evaluation and investigations. The safety profile of BNT162b2 is currently only available from short-term clinical studies. Less common adverse effects of BNT162b2 have been reported, including pericarditis, arrhythmia, deep-vein thrombosis, pulmonary embolism, myocardial infarction, intracranial hemorrhage, and thrombocytopenia [4,9,10,11,12,13,14,15,16,17,18,19,20]. There are also studies that report adverse effects observed in other types of vaccines [21,22,23,24]. To better understand mechanisms underlying vaccine-related adverse effects, clinical investigations as well as cellular and molecular analyses are needed.

A recent study showed that SARS-CoV-2 RNAs can be reverse-transcribed and integrated into the genome of human cells [25]. This gives rise to the question of if this may also occur with BNT162b2, which encodes partial SARS-CoV-2 RNA. In pharmacokinetics data provided by Pfizer to European Medicines Agency (EMA), BNT162b2 biodistribution was studied in mice and rats by intra-muscular injection with radiolabeled LNP and luciferase modRNA. Radioactivity was detected in most tissues from the first time point (0.25 h), and results showed that the injection site and the liver were the major sites of distribution, with maximum concentrations observed at 8–48 h post-dose [26]. Furthermore, in animals that received the BNT162b2 injection, reversible hepatic effects were observed, including enlarged liver, vacuolation, increased gamma glutamyl transferase (γGT) levels, and increased levels of aspartate transaminase (AST) and alkaline phosphatase (ALP) [26]. Transient hepatic effects induced by LNP delivery systems have been reported previously [27,28,29,30], nevertheless, it has also been shown that the empty LNP without modRNA alone does not introduce any significant liver injury [27]. Therefore, in this study, we aim to examine the effect of BNT162b2 on a human liver cell line in vitro and investigate if BNT162b2 can be reverse transcribed into DNA through endogenous mechanisms.

## 2. Materials and Methods

### 2.1. Cell Culture

Huh7 cells (JCRB Cell Bank, Osaka, Japan) were cultured in 37 °C at 5% CO_2_ with DMEM medium (HyClone, HYCLSH30243.01) supplemented with 10% (*v*/*v*) fetal bovine serum (Sigma-Aldrich, F7524-500ML, Burlington, MA, USA) and 1% (*v*/*v*) Penicillin-Streptomycin (HyClone, SV30010, Logan, UT, USA). For BNT162b2 treatment, Huh7 cells were seeded with a density of 200,000 cells/well in 24-well plates. BNT162b2 mRNA vaccine (Pfizer BioNTech, New York, NY, USA) was diluted with sterile 0.9% sodium chloride injection, USP into a final concentration of 100 μg/mL as described in the manufacturer’s guideline [31]. BNT162b2 suspension was then added in cell culture media to reach final concentrations of 0.5, 1.0, or 2.0 μg/mL. Huh7 cells were incubated with or without BNT162b2 for 6, 24, and 48 h. Cells were washed thoroughly with PBS and harvested by trypsinization and stored in −80 °C until further use.

### 2.2. REAL-TIME RT-QPCR

RNA from the cells was extracted with RNeasy Plus Mini Kit (Qiagen, 74134, Hilden, Germany) following the manufacturer’s protocol. RT-PCR was performed using RevertAid First Strand cDNA Synthesis kit (Thermo Fisher Scientific, K1622, Waltham, MA, USA) following the manufacturers protocol. Real-time qPCR was performed using Maxima SYBR Green/ROX qPCR Master Mix (Thermo Fisher Scientific, K0222, Waltham, MA, USA) with primers for BNT162b2, *LINE-1* and housekeeping genes *ACTB* and *GAPDH* (Table 1).

### 2.3. Immunofluorescence Staining and Confocal Imaging

Huh7 cells were cultured in eight-chamber slides (LAB-TEK, 154534, Santa Cruz, CA, USA) with a density of 40,000 cells/well, with or without BNT162b2 (0.5, 1 or 2 µg/mL) for 6 h. Immunohistochemistry was performed using primary antibody anti-LINE-1 ORF1p mouse monoclonal antibody (Merck, 3574308, Kenilworth, NJ, USA), secondary antibody Cy3 Donkey anti-mouse (Jackson ImmunoResearch, West Grove, PA, USA), and Hoechst (Life technologies, 34850, Carlsbad, CA, USA), following the protocol from Thermo Fisher (Waltham, MA, USA). Two images per condition were taken using a Zeiss LSM 800 and a 63X oil immersion objective, and the staining intensity was quantified on the individual whole cell area and the nucleus area on 15 cells per image by ImageJ 1.53c. LINE-1 staining intensity for the cytosol was calculated by subtracting the intensity of the nucleus from that of the whole cell. All images of the cells were assigned a random number to prevent bias. To mark the nuclei (determined by the Hoechst staining) and the whole cells (determined by the borders of the LINE-1 fluorescence), the Freehand selection tool was used. These areas were then measured, and the mean intensity was used to compare the groups.

### 2.4. Genomic DNA Purification, PCR Amplification, Agarose Gel Purification, and Sanger Sequencing

Genomic DNA was extracted from cell pellets with PBND buffer (10 mM Tris-HCl pH 8.3, 50 mM KCl, 2.5 mM MgCl2, 0.45% NP-40, 0.45% Tween-20) according to protocol described previously [32]. To remove residual RNA from the DNA preparation, RNase (100 µg/mL, Qiagen, Hilden, Germany) was added to the DNA preparation and incubated at 37 °C for 3 h, followed by 5 min at 95 °C. PCR was then performed using primers targeting BNT162b2 (sequences are shown in Table 1), with the following program: 5 min at 95 °C, 35 cycles of 95 °C for 30 s, 58 °C for 30 s, and 72 °C for 1 min; finally, 72 °C for 5 min and 12 °C for 5 min. PCR products were run on 1.4% (*w*/*v*) agarose gel. Bands corresponding to the amplicons of the expected size (444 bps) were cut out and DNA was extracted using QIAquick PCR Purification Kit (Qiagen, 28104, Hilden, Germany), following the manufacturer’s instructions. The sequence of the DNA amplicon was verified by Sanger sequencing (Eurofins Genomics, Ebersberg, Germany).

#### Statistics

Statistical comparisons were performed using two-tailed Student’s *t*-test and ANOVA. Data are expressed as the mean ± SEM or ± SD. Differences with *p* < 0.05 are considered significant.

### 2.5. Ethical Statements

The Huh7 cell line was obtained from Japanese Collection of Research Bioresources (JCRB) Cell Bank.

## 3. Results

### 3.1. BNT162b2 Enters Human Liver Cell Line Huh7 Cells at High Efficiency

**Figure 1:**
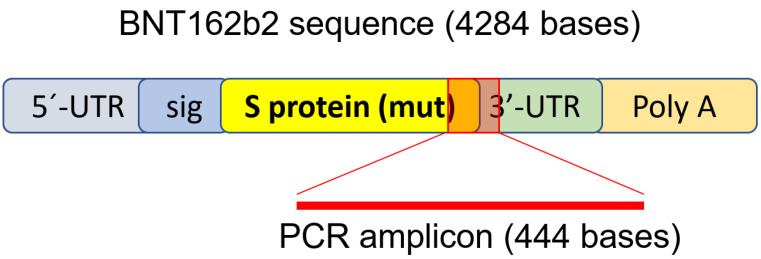
PCR primer set used to detect mRNA level and reverse-transcription of BNT162b2. Illustration of BNT162b2 was adapted from previously described literature [34].

To determine if BNT162b2 enters human liver cells, we exposed human liver cell line Huh7 to BNT162b2. In a previous study on the uptake kinetics of LNP delivery in Huh7 cells, the maximum biological efficacy of LNP was observed between 4–7 h [33]. Therefore, in our study, Huh7 cells were cultured with or without increasing concentrations of BNT162b2 (0.5, 1.0 and 2.0 µg/mL) for 6, 24, and 48 h. RNA was extracted from cells and a real-time quantitative reverse transcription polymerase chain reaction (RT-qPCR) was performed using primers targeting the BNT162b2 sequence, as illustrated in Figure 1. The full sequence of BNT162b2 is publicly available [34] and contains a two-nucleotides cap; 5′- untranslated region (UTR) that incorporates the 5′ -UTR of a human α-globin gene; the full-length of SARS-CoV-2 S protein with two proline mutations; 3′-UTR that incorporates the human mitochondrial 12S rRNA (mtRNR1) segment and human AES/TLE5 gene segment with two C→U mutations; poly(A) tail. Detailed analysis of the S protein sequence in BNT162b2 revealed 124 sequences that are 100% identical to human genomic sequences and three sequences with only one nucleotide (nt) mismatch in 19–26 nts (Table S1, see Supplementary Materials). To detect BNT162b2 RNA level, we designed primers with forward primer located in SARS-CoV-2 S protein regions and reverse primer in 3′-UTR, which allows detection of PCR amplicon unique to BNT162b2 without unspecific binding of the primers to human genomic regions.

**Figure 2:**
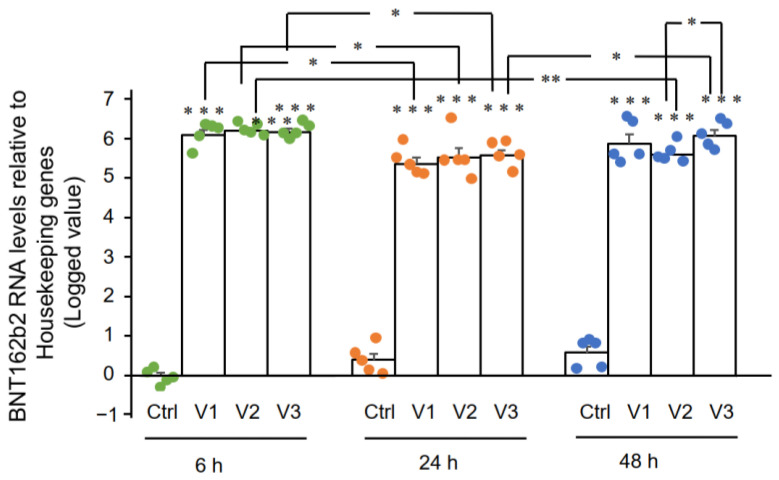
BNT162b2 mRNA levels in Huh7 cells treated with BNT162b2. Huh7 cells were treated without (Ctrl) or with 0.5 (V1), 1 (V2), and 2 µg/mL (V3) of BNT162b2 for 6 (green dots), 24 (orange dots), and 48 h (blue dots). RNA was purified and qPCR was performed using primers targeting BNT162b2. RNA levels of BNT162b2 are presented as logged 2^−ΔΔCT^ values relative to house-keeping genes *GAPDH* and *ACTB*. Results are from five independent experiments (*n* = 5). Differences between respective groups were analyzed using two-tailed Student’s *t*-test. Data are expressed as the mean ± SEM. (* *p* < 0.05; ** *p* < 0.01; *** *p* < 0.001 vs. respective control at each time point, or as indicated).

RT-qPCR results showed that Huh7 cells treated with BNT162b2 had high levels of BNT162b2 mRNA relative to housekeeping genes at 6, 24, and 48 h (Figure 2, presented in logged 2^−ΔΔCT^ due to exceptionally high levels). The three BNT162b2 concentrations led to similar intracellular BNT162b2 mRNA levels at the different time points, except that the significant difference between 1.0 and 2.0 µg/mL was observed at 48 h. BNT162b2 mRNA levels were significantly decreased at 24 h compared to 6 h, but increased again at 48 h.

### 3.2. Effect of BNT162b2 on Human Endogenous Reverse Transcriptase Long Interspersed Nuclear Element-1 (LINE-1)

**Figure 3:**
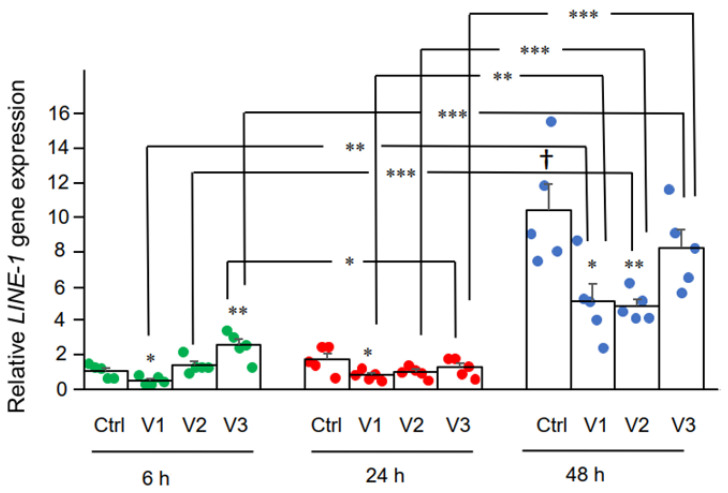
*LINE-1* mRNA levels in Huh7 cells treated with BNT162b2. Huh7 cells were treated without (Ctrl) or with 0.5 (V1), 1 (V2), and 2 µg/mL (V3) of BNT162b2 for 6 (green dots), 24 (red dots), and 48 h (blue dots). RNA was purified and qPCR was performed using primers targeting *LINE-1*. RNA levels of *LINE-1* are presented as 2^−ΔΔCT^ values relative to house-keeping genes *GAPDH* and *ACTB*. Results are from five independent experiments (*n* = 5). Differences between respective groups were analyzed using two-tailed Student’s *t*-test. Data are expressed as the mean ± SEM. (* *p* < 0.05; ** *p* < 0.01; *** *p* < 0.001 vs. respective control at each time point, or as indicated; † *p* < 0.05 vs. 6 h-Ctrl).

Here we examined the effect of BNT162b2 on *LINE-1* gene expression. RT-qPCR was performed on RNA purified from Huh7 cells treated with BNT162b2 (0, 0.5, 1.0, and 2.0 µg/mL) for 6, 24, and 48 h, using primers targeting *LINE-1*. Significantly increased *LINE-1* expression compared to control was observed at 6 h by 2.0 µg/mL BNT162b2, while lower BNT162b2 concentrations decreased *LINE-1* expression at all time points (Figure 3).

**Figure 4:**
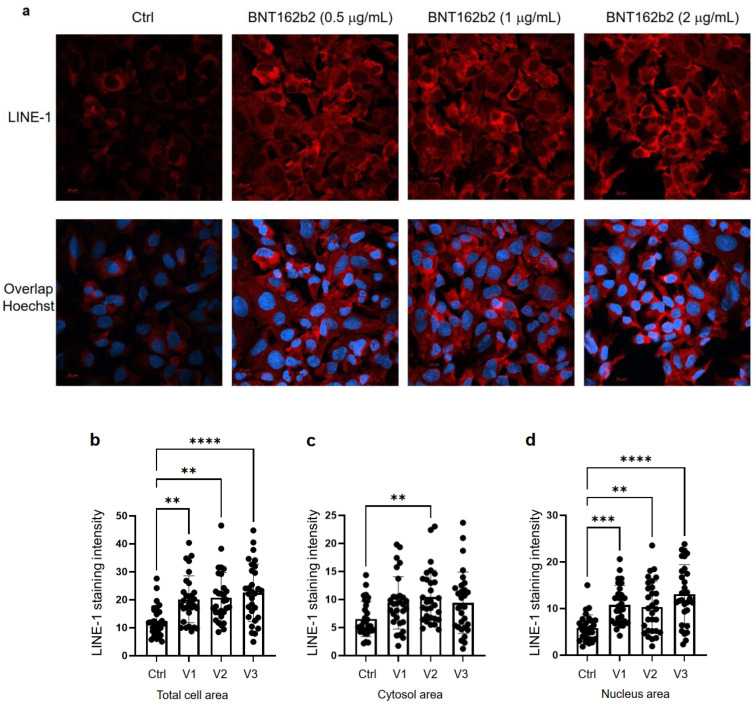
Immunohistochemistry of Huh7 cells treated with BNT162b2 on LINE-1 protein distribution. Huh7 cells were treated without (Ctrl) or with 0.5, 1, and 2 µg/mL of BNT162b2 for 6 h. Cells were fixed and stained with antibodies binding to LINE-1 ORF1p (red) and DNA-specific probe Hoechst for visualization of cell nucleus (blue). (**a**) Representative images of LINE-1 expression in Huh7 cells treated with or without BNT162b2. (**b**–**d**) Quantification of LINE-1 protein in whole cell area (**b**), cytosol (**c**), and nucleus (**d**). All data were analyzed using One-Way ANOVA, and graphs were created using GraphPad Prism V 9.2. All data is presented as mean ± SD (** *p* < 0.01; *** *p* < 0.001; **** *p* < 0.0001 as indicated).

Next, we studied the effect of BNT162b2 on LINE-1 protein level. The full-length LINE-1 consists of a 5′ untranslated region (UTR), two open reading frames (ORFs), ORF1 and ORF2, and a 3′UTR, of which ORF1 is an RNA binding protein with chaperone activity. The retrotransposition activity of LINE-1 has been demonstrated to involve ORF1 translocation to the nucleus [35]. Huh7 cells treated with or without BNT162b2 (0.5, 1.0 and 2.0 µg/mL) for 6 h were fixed and stained with antibodies binding to LINE-1 ORF1p, and DNA-specific probe Hoechst for visualization of cell nucleus (Figure 4a). Quantification of immunofluorescence staining intensity showed that BNT162b2 increased LINE-1 ORF1p protein levels in both the whole cell area and nucleus at all concentrations tested (Figure 4b–d).

### 3.3. Detection of Reverse Transcribed BNT162b2 DNA in Huh7 Cells

**Figure 5:**
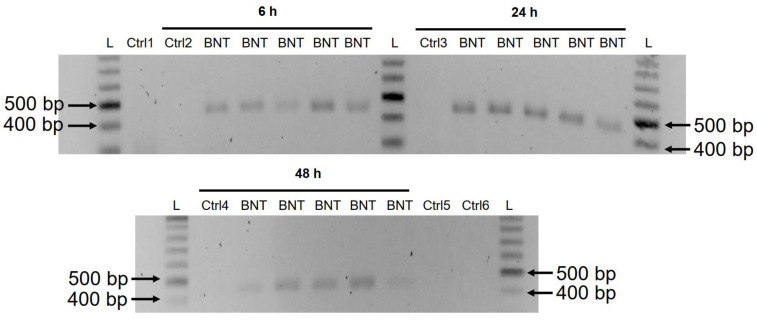
Detection of DNA amplicons of BNT162b2 in Huh7 cells treated with BNT162b2. Huh7 cells were treated without (Ctrl) or with 0.5 µg/mL of BNT162b2 for 6, 24, and 48 h. Genomic DNA was purified and digested with 100 µg/mL RNase. PCR was run on all samples with primers targeting BNT162b2, as shown in Figure 1 and Table 1. DNA amplicons (444 bps) were visualized on agarose gel. BNT: BNT162b2; L: DNA ladder; Ctrl1: cultured Huh7 cells; Ctrl2: Huh7 cells without BNT162b2 treatment collected at 6 h; Ctrl3: Huh7 cells without BNT162b2 treatment collected at 24 h; Ctrl4: Huh7 cells without BNT162b2 treatment collected at 48 h; Ctrl5: RNA from Huh7 cells treated with 0.5 µg/mL of BNT162b2 for 6 h; Ctrl6: RNA from Huh7 cells treated with 0.5 µg/mL of BNT162b2 for 6 h, digested with RNase.

A previous study has shown that entry of LINE-1 protein into the nucleus is associated with retrotransposition [35]. In the immunofluorescence staining experiment described above, increased levels of LINE-1 in the nucleus were observed already at the lowest concentration of BNT162b2 (0.5 µg/mL). To examine if BNT162b2 is reversely transcribed into DNA when LINE-1 is elevated, we purified genomic DNA from Huh7 cells treated with 0.5 µg/mL of BNT162b2 for 6, 24, and 48 h. Purified DNA was treated with RNase to remove RNA and subjected to PCR using primers targeting BNT162b2, as illustrated in Figure 1. Amplified DNA fragments were then visualized by electrophoresis and gel-purified (Figure 5). BNT162b2 DNA amplicons were detected in all three time points (6, 24, and 48 h). Sanger sequencing confirmed that the DNA amplicons were identical to the BNT162b2 sequence flanked by the primers (Table 2). To ensure that the DNA amplicons were derived from DNA but not BNT162b2 RNA, we also performed PCR on RNA purified from Huh7 cells treated with 0.5 µg/mL BNT162b2 for 6 h, with or without RNase treatment (Ctrl 5 and 6 in Figure 5), and no amplicon was detected in the RNA samples subjected to PCR.

## 4. Discussion

In this study we present evidence that COVID-19 mRNA vaccine BNT162b2 is able to enter the human liver cell line Huh7 in vitro. BNT162b2 mRNA is reverse transcribed intracellularly into DNA as fast as 6 h after BNT162b2 exposure. A possible mechanism for reverse transcription is through endogenous reverse transcriptase LINE-1, and the nucleus protein distribution of LINE-1 is elevated by BNT162b2.

Intracellular accumulation of LNP in hepatocytes has been demonstrated in vivo [36]. A preclinical study on BNT162b2 showed that BNT162b2 enters the human cell line HEK293T cells and leads to robust expression of BNT162b2 antigen [37]. Therefore, in this study, we first investigated the entry of BNT162b2 in the human liver cell line Huh7 cells. The choice of BNT162b2 concentrations used in this study warrants explanation. BNT162b2 is administered as a series of two doses three weeks apart, and each dose contains 30 µg of BNT162b2 in a volume of 0.3 mL, which makes the local concentration at the injection site at the highest 100 µg/mL [31]. A previous study on mRNA vaccines against H10N8 and H7N9 influenza viruses using a similar LNP delivery system showed that the mRNA vaccine can distribute rather nonspecifically to several organs such as liver, spleen, heart, kidney, lung, and brain, and the concentration in the liver is roughly 100 times lower than that of the intra-muscular injection site [38]. In the assessment report on BNT162b2 provided to EMA by Pfizer, the pharmacokinetic distribution studies in rats demonstrated that a relatively large proportion (up to 18%) of the total dose distributes to the liver [26]. We therefore chose to use 0.5, 1, and 2 μg/mL of vaccine in our experiments on the liver cells. However, the effect of a broader range of lower and higher concentrations of BNT162b2 should also be verified in future studies.

In the current study, we employed a human liver cell line for in vitro investigation. It is worth investigating if the liver cells also present the vaccine-derived SARS-CoV-2 spike protein, which could potentially make the liver cells targets for previously primed spike protein reactive cytotoxic T cells. There has been case reports on individuals who developed autoimmune hepatitis [39] after BNT162b2 vaccination. To obtain better understanding of the potential effects of BNT162b2 on liver function, in vivo models are desired for future studies.

In the BNT162b2 toxicity report, no genotoxicity nor carcinogenicity studies have been provided [26]. Our study shows that BNT162b2 can be reverse transcribed to DNA in liver cell line Huh7, and this may give rise to the concern if BNT162b2-derived DNA may be integrated into the host genome and affect the integrity of genomic DNA, which may potentially mediate genotoxic side effects. At this stage, we do not know if DNA reverse transcribed from BNT162b2 is integrated into the cell genome. Further studies are needed to demonstrate the effect of BNT162b2 on genomic integrity, including whole genome sequencing of cells exposed to BNT162b2, as well as tissues from human subjects who received BNT162b2 vaccination.

Human autonomous retrotransposon LINE-1 is a cellular endogenous reverse transcriptase and the only remaining active transposon in humans, able to retrotranspose itself and other nonautonomous elements [40,41], and ~17% of the human genome are comprised of LINE-1 sequences [42]. The nonautonomous *Alu* elements, short, interspersed nucleotide elements (SINEs), variable-number-of-tandem-repeats (VNTR), as well as cellular mRNA-processed pseudogenes, are retrotransposed by the LINE-1 retrotransposition proteins working in *trans* [43,44]. A recent study showed that endogenous LINE-1 mediates reverse transcription and integration of SARS-CoV-2 sequences in the genomes of infected human cells [25]. Furthermore, expression of endogenous LINE-1 is often increased upon viral infection, including SARS-CoV-2 infection [45,46,47]. Previous studies showed that LINE-1 retrotransposition activity is regulated by RNA metabolism [48,49], DNA damage response [50], and autophagy [51]. Efficient retrotransposition of LINE-1 is often associated with cell cycle and nuclear envelope breakdown during mitosis [52,53], as well as exogenous retroviruses [54,55], which promotes entrance of LINE-1 into the nucleus. In our study, we observed increased LINE-1 ORF1p distribution as determined by immunohistochemistry in the nucleus by BNT162b2 at all concentrations tested (0.5, 1, and 2 μg/mL), while elevated *LINE-1* gene expression was detected at the highest BNT162b2 concentration (2 μg/mL). It is worth noting that gene transcription is regulated by chromatin modifications, transcription factor regulation, and the rate of RNA degradation, while translational regulation of protein involves ribosome recruitment on the initiation codon, modulation of peptide elongation, termination of protein synthesis, or ribosome biogenesis. These two processes are controlled by different mechanisms, and therefore they may not always show the same change patterns in response to external challenges. The exact regulation of LINE-1 activity in response to BNT162b2 merits further study.

The cell model that we used in this study is a carcinoma cell line, with active DNA replication which differs from non-dividing somatic cells. It has also been shown that Huh7 cells display significant different gene and protein expression including upregulated proteins involved in RNA metabolism [56]. However, cell proliferation is also active in several human tissues such as the bone marrow or basal layers of epithelia as well as during embryogenesis, and it is therefore necessary to examine the effect of BNT162b2 on genomic integrity under such conditions. Furthermore, effective retrotransposition of LINE-1 has also been reported in non-dividing and terminally differentiated cells, such as human neurons [57,58].

The Pfizer EMA assessment report also showed that BNT162b2 distributes in the spleen (<1.1%), adrenal glands (<0.1%), as well as low and measurable radioactivity in the ovaries and testes (<0.1%) [26]. Furthermore, no data on placental transfer of BNT162b2 is available from Pfizer EMA assessment report. Our results showed that BNT162b2 mRNA readily enters Huh7 cells at a concentration (0.5 µg/mL) corresponding to 0.5% of the local injection site concentration, induce changes in LINE-1 gene and protein expression, and within 6 h, reverse transcription of BNT162b2 can be detected. It is therefore important to investigate further the effect of BNT162b2 on other cell types and tissues both in vitro and in vivo.

## 5. Conclusions

Our study is the first in vitro study on the effect of COVID-19 mRNA vaccine BNT162b2 on human liver cell line. We present evidence on fast entry of BNT162b2 into the cells and subsequent intracellular reverse transcription of BNT162b2 mRNA into DNA.

## Figures and Tables

**Table 1:** Primer sequences of RT-qPCR and PCR.

Target	Sequence
*ACTB* forward	CCTCGCCTTTGCCGATCC
*ACTB* reverse	GGATCTTCATGAGGTAGTCAGTC
*GAPDH* forward	CTCTGCTCCTCCTGTTCGAC
*GAPDH* reverse	TTAAAAGCAGCCCTGGTGAC
*LINE-1* forward	TAACCAATACAGAGAAGTGC
*LINE-1* reverse	GATAATATCCTGCAGAGTGT
BNT162b2 forward	CGAGGTGGCCAAGAATCTGA
BNT162b2 reverse	TAGGCTAAGCGTTTTGAGCTG

**Table 2:** Sanger sequencing result of the BNT162b2 amplicon.

CGAGGTGGCCAAGAATCTGAACGAGAGCCTGATCGACCTGCAAGAACTGGGGAAGT ACGAGCAGTACATCAAGTGGCCCTGGTACATCTGGCTGGGCTTTATCGCCGGACTGATTG CCATCGTGATGGTCACAATCATGCTGTGTTGCATGACCAGCTGCTGTAGCTGCCTGAAGG GCTGTTGTAGCTGTGGCAGCTGCTGCAAGTTCGACGAGGACGATTCTGAGCCCGTGCTGA
AGGGCGTGAAACTGCACTACACATGATGACTCGAGCTGGTACTGCATGCACGCAATGCTA GCTGCCCCTTTCCCGTCCTGGGTACCCCGAGTCTCCCCCGACCTCGGGTCCCAGGTATGC TCCCACCTCCACCTGCCCCACTCACCACCTCTGCTAGTTCCAGACACCTCCCAAGCACGC AGCAATGCAGCTCAAAACGCTTAGCCTA

## Data Availability

All data supporting the findings of this study are available within the article and supporting information.

## Supplementary Materials

The following supporting information can be downloaded at: https://www.mdpi.com/article/10.3390/cimb44030073/s1.
